# Network Pharmacology-Based Strategy for the Investigation of the Anti-Osteoporosis Effects and Underlying Mechanism of Zhuangguguanjie Formulation

**DOI:** 10.3389/fphar.2021.727808

**Published:** 2021-10-01

**Authors:** Wang Gong, Xingren Chen, Tianshu Shi, Xiaoyan Shao, Xueying An, Jianghui Qin, Xiang Chen, Qing Jiang, Baosheng Guo

**Affiliations:** ^1^ Department of Sports Medicine and Adult Reconstructive Surgery, The Affiliated Hospital of Nanjing University Medical School and State Key Laboratory of Pharmaceutical Biotechnology, Medical School, Nanjing Drum Tower Hospital, Nanjing University, Nanjing, China; ^2^ Jiangsu Key Laboratory of Molecular Medicine, Medical School, Nanjing University, Nanjing, China; ^3^ Laboratory for Bone and Joint Disease, Model Animal Research Center (MARC), Medical School, Nanjing University, Nanjing, China

**Keywords:** osteoporosis, Zhuangguguanjie, network pharmacology, PI3K/AKT, mTOR/S6K1

## Abstract

As the society is aging, the increasing prevalence of osteoporosis has generated huge social and economic impact, while the drug therapy for osteoporosis is limited due to multiple targets involved in this disease. Zhuangguguanjie formulation (ZG) is extensively used in the clinical treatment of bone and joint diseases, but the underlying mechanism has not been fully described. This study aimed to examine the therapeutic effect and potential mechanism of ZG on postmenopausal osteoporosis. The ovariectomized (OVX) mice were treated with normal saline or ZG for 4 weeks after ovariectomy following a series of analyses. The bone mass density (BMD) and trabecular parameters were examined by micro-CT. Bone remodeling was evaluated by the bone histomorphometry analysis and ELISA assay of bone turnover biomarkers in serum. The possible drug–disease common targets were analyzed by network pharmacology. To predict the potential biological processes and related pathways, GO/KEGG enrichment analysis was performed. The effects of ZG on the differentiation phenotype of osteoclasts and osteoblasts and the predicted pathway were verified *in vitro*. The results showed that ZG significantly improved the bone mass and micro-trabecular architecture in OVX mice compared with untreated OVX mice. ZG could promote bone formation and inhibit bone resorption to ameliorate ovariectomy-induced osteoporosis as evidenced by increased number of osteoblast (N.Ob/Tb.Pm) and decreased number of osteoclast (N.Oc/Tb.Pm) in treated group compared with untreated OVX mice. After identifying potential drug–disease common targets by network pharmacology, GO enrichment analysis predicted that ZG might affect various biological processes including osteoblastic differentiation and osteoclast differentiation. The KEGG enrichment analysis suggested that PI3K/Akt and mTOR signaling pathways could be the possible pathways. Furthermore, the experiments *in vitro* validated our findings. ZG significantly down-regulated the expression of osteoclast differentiation markers, reduced osteoclastic resorption, and inhibited the phosphorylation of PI3K/Akt, while ZG obviously up-regulated the expression of osteogenic biomarkers, promoted the formation of calcium nodules, and hampered the phosphorylation of 70S6K1/mTOR, which can be reversed by the corresponding pathway activator. Thus, our study suggested that ZG could inhibit the PI3K/Akt signaling pathway to reduce osteoclastic bone resorption as well as hamper the mTORC1/S6K1 signaling pathway to promote osteoblastic bone formation.

## Introduction

Osteoporosis has been defined as a systemic skeletal disease characterized by reduced bone mineral density and micro-architecture deterioration (Weitzmann and Ofotokun, 2016), with an increase in susceptibility to fractures (Fuggle et al., 2019). The prevalence of osteoporosis is extremely high in older population (10.4% for males, 31.2% for females) (Zhu et al., 2010). The search for anti-osteoporosis medications still remains a challenge. Currently, standard antiresorptive therapeutic schedules for osteoporosis mainly include bisphosphonate and estrogen replacement therapy (Gatti and Fassio, 2019) and were restricted by single target and adverse effects (Khosla and Hofbauer, 2017). Anabolic therapies have been approved to protect osteoporosis *via* facilitating bone formation alone or with antiresorptive agents (Estell and Rosen, 2021). However, there is no proposed curative treatment for osteoporosis that could simultaneously inhibit bone resorption and promote bone formation.

Recently, traditional Chinese medicine (TCM) has been widely applied due to significant therapeutic efficiency and fewer adverse effects (Chao et al., 2017). Zhuangguguanjie formulation (ZG) is a classical TCM consisted of twelve Chinese medicinal herbs. It is widely used clinically to treat bone and joint diseases (Zhang XL. et al., 2016). Previous study has confirmed that ZG could protect articular cartilage *via* the p-Akt/Caspase 3 pathway (Lu et al., 2018). ZG has also been reported to treat osteoporosis with less adverse effects (Liu et al., 2014). Some components of ZG such as icariin and psoralen have been reported that could attenuate osteoclast differentiation or promote osteoblast functions to cure osteoporosis. Icariin could promote osteogenic differentiation of BMSCs through the Wnt/β-Catenin signaling pathway (Gao et al., 2021) and inhibit osteoclastogenic differentiation *via* the MAPK pathway (Xu et al., 2019). Psoralen is reported to decrease osteoclast differentiation and bone resorption *via* inhibition of the AKT pathway (Chai et al., 2018). However, the dosage of single components used in clinical treatment still brings some side effects compared with ZG formulation (Indran et al., 2016).

Despite intensive clinical application and research, the function and mechanism of ZG on osteoporosis have not been fully investigated. As a systematic biological method, network pharmacology provides a network visualization of components and targets that are applied for the comprehensive study of TCM compounds (WANG et al., 2021). In this study, we examined the effect of ZG on osteoporosis in ovariectomized (OVX) mice. Both network pharmacology and experiments *in vitro/vivo* were used to unravel the underlying mechanisms.

## Materials and Methods

### Reagents

Traditional Chinese medicine ZG was obtained from Sanjiu Medical & Pharmaceutical Co., Ltd. (Beijing, China). It was dissolved in normal saline as a vehicle for animal experiment while using phosphate-buffered saline (PBS) as vehicle for cell experiment. Fetal bovine serum (FBS) was purchased from Gibco (United States). Alpha-minimal essential medium (a-MEM), PBS, trypsin−EDTA, penicillin, and streptomycin were purchased from Wisent (Canada). Cell Counting Kit-8 (CCK-8) and MHY1485 were from MCE (China). IGF-1, M-CSF, RANKL were from R&D systems (United States). Osteogenic Differentiation Medium and Alizarin Red staining kit were purchased from Cyagen (China).

### LC-MS/MS and HPLC Analysis

LC-MS/MS analysis was performed using Thermo QE HF-X mass spectrometer and Thermo U3000 liquid chromatography. The analysis was conducted using Waters, ACQUITY UPLC HSS T3 1.7 µm, 2.1 mm× 150 mm column. The mobile phase A consisted of 5 mM ammonium formate in H_2_O, mobile phase B consisted of acetonitrile, mobile phase C consisted of 0.1% formic acid in H_2_O, mobile phase D consisted of 0.1% formic acid in acetonitrile. The liquid chromatography was carried out using a gradient program at a flow rate of 0.4 ml/min; the injection volume was 10 μl; the column oven temperature was 40°C. The acquisition of high-resolution mass spectra was conducted in the positive and negative ion modes. Optimized ionization conditions were as follows: sheath gas flow, 8 L/min; capillary voltage, 2,500 V; gas temperature, 300°C; the analysis was carried out using a scan from 80 to 1,000 m/z. Identification of metabolites was conducted by searching the OCTML database according to exact mass matching (<10 ppm) and secondary spectrum matching. HPLC was performed to determine the content of psoralen, isopsoralen, icariin, and osthole. The ZG extract was dissolved to a final concentration of 20 mg/ml in methyl alcohol. The diluted solution was passed through a 0.45 µM filter membrane prior to HPLC analysis. The wavelength of UV detection was set at 254 nm for psoralen, isopsoralen, and icariin, while was set at 320 nm for osthole.

### Animals

Eight-week-old male C57BL/6 mice (weighing 20 ± 2 g) were fed under pathogen-free conditions, supplied by the Model Animal Research Center of Nanjing University. The animals were maintained in a 12-h light/dark cycle. A total of 36 mice were equally divided into the following groups: sham with vehicle treatment group, ovariectomy with vehicle treatment group, and ovariectomy with ZG treatment group, 12 mice used per group. Subsequently, the OVX group and the OVX + ZG group underwent ovariectomy, anesthetized by isoflurane. A similar incision was made to the sham group, which was subsequently sutured, without any other procedures. All mice were monitored on a daily basis for postoperative complications within the first week after surgery. Both saline and ZG (468 mg/kg/day) administration started at 4 weeks after surgery. The mice were sacrificed by isoflurane overdose following 4 weeks of continuous treatment. After sacrifice, the bilateral femora were isolated and collected, as well as blood serum. The experimental protocols were reviewed and approved by the Animal Care Committee of the Affiliate Drum Tower Hospital, Medical school of Nanjing University in accordance with the Institutional Animal Care and Use Committee guidelines (2021AE01031).

### Morphometric and Histological Assessments

Femurs and tibias were fixed in 4% paraformaldehyde for 24 h immediately after death. The fixed samples were imaged by micro-CT (vivaCT80, SCANCO Medical AG, Switzerland). To analyze the trabecular bone, a region of interest was selected 0.5 mm below the growth plate of the femur and tibia. Bone mineral density (BMD), bone volume to total volume (BV/TV), trabecular number (Tb. N), trabecular separation (Tb. Sp), and trabecular thickness (Tb. Th) were analyzed with the built-in software. After micro-CT analyses, femurs were decalcified with 5% EDTA and were embedded in paraffin blocks, and 5 mm sections were cut in serial incisions in the coronal plane. The sections were stained with hematoxylin and eosin (HE), and then TRAP staining was used to detect osteoclast activity. The expression of osteocalcin was examined by immunohistochemistry. Briefly, tissue sections were treated with 3% H_2_O_2_ to remove endogenous peroxidase, incubated with anti-osteocalcin antibody (ab93876, Abcam, United Kingdom) at 4°C overnight prior to addition of secondary antibody (AS014, ABclonal, China), incubated at 37°C for 120 min, then colored by diaminobenzidine (DAB) reaction staining. Finally, sections were evaluated by microscopy (BX53, Olympus) in a blinded manner. Quantitative analyses were performed using ImageJ Software.

### Xylenol Orange-Calcein Double Labeling Analyses

Mice were injected with calcein (10 mg/kg) at 10 days before euthanasia and xylenol orange (30 mg/kg) at 3 days before euthanasia. The femurs were collected and prepared for hard tissue sections with EXAKT 300CP. Fluorescence images were then obtained using confocal microscope (FV3000, Olympus).

### Serum Biochemistry

A total of 500 μl of blood was collected from the hearts of the mice following anesthesia. Serum samples were obtained by centrifugation of blood at 3,000 rpm for 15 min, and they were stored at −80°C prior to analysis. An ELISA assay was used to measure the plasma concentration of the biomarkers PINP, CTX-1, and TNF-α (Cloud Clone Corp, China) according to the manufacturer’s instructions. The optical absorbance at 450 nm was determined using a microplate absorbance reader (Model 680 Microplate Reader, Bio-Rad).

### Screening for Potential Targets of Zhuangguguanjie Formulation

All components in ZG were searched from the Traditional Chinese Medicine Systems Pharmacology Database and Analysis Platform (TCMSP) and Bioinformatics Analysis Tool for Molecular mechanism (BATMAN). Then related chemical compositions were supplemented according to literature. All results were saved in SDF format. In the Swiss Target Prediction database, species was restricted to “Homo sapiens”, and the candidate targets with prediction scores >0 were selected as the potential targets of ZG.

### Potential Targets Intersection of Zhuangguguanjie Formulation With Osteoporosis

Disease targets were retrieved using “osteoporosis” as the keywords based on the Disgenet database. All drug targets and disease targets were introduced into Venny2.1.0 to obtain common targets of ZG and osteoporosis. Cytoscape (version 3.2.1) was used to establish the drug-bioactive ingredient-target-disease network of ZG acting on osteoporosis. Finally, we used Network Analyzer function to analyze the main active components of traditional Chinese medicine compound prescription.

### Protein–Protein Interaction Network Construction

The 212 common targets were uploaded to the STRING database to obtain the network relation of target interaction, with minimum required interaction score>0.4. All data were imported in Cytoscape for constructing the PPI network, in which the color and size of nodes represent the degree of the node.

### Enrichment Analysis of GO and KEGG Pathways

To convert the drug–disease common target into entrez ID, we installed the Bioconductor package “org.Hs.eg.db” in R software and ran it. After that, the “cluster Profiler” package was installed in R software, and enrichment analysis of GO and KEGG was carried out according to the transformed entrez ID, with *p* < 0.05 and Q < 0.05. The results were output in the form of bar chart and bubble chart, respectively.

### CCK-8-Based Cytotoxicity Assay

The cytotoxicity of ZG was determined by CCK-8 assay. Briefly, the bone marrow monocytes (BMMs) and preosteoblasts were seeded onto 96-well plates independently at a concentration of 1 × 10^4^ cells/ml and cultured until they adhered completely. Then, cells were treated with ZG at different concentrations (0 µg/ml, 30 µg/ml, 60 µg/ml, 90 µg/ml, 180 µg/ml, 360 µg/ml, 720 µg/ml) for 24 h. After that, 10 µl of CCK-8 was added and incubated for 1 h. The absorbance was recorded at 450 nm, and experiments were performed in triplicate and repeated three times independently.

### *In Vitro* Osteoclastogenesis Assay

BMMs were isolated from 4-week-old C57/BL6 male mice by separating the long bones and rinsing the medullary cavity. BMMs were cultured in a-MEM supplemented with 10% FBS, 1% penicillin-streptomycin solution, and 30 ng/ml M-CSF. After 48 h, the cell medium was removed, and the adherent BMMs were considered as osteoclast precursor cells that used for subsequent experiments. Then BMMs were seeded in 96-well plates and cultured to100% confluence. Osteoclast formation was induced through medium replacement with a-MEM containing 30 ng/ml M-CSF and 50 ng/ml RANKL, with different concentrations of ZG (0 µg/ml, 90 µg/ml, 180 µg/ml) and IGF-1 (0 ng/ml, 10 ng/ml) depending on experimental design. After 10 days, the cells were fixed in 4% PFA and stained for TRAP using an acid phosphatase leucocyte staining kit (Sigma-Aldrich), according to the instructions. To study the actin ring formation, the cells were permeabilized with 0.3% Triton-X 100 and cultured with phalloidin to visualize F-actin after fixed in 4% PFA. Both TRAP staining and phalloidin staining were photoed by a fluorescence microscope (Ti2 U, Nikon).

### *In Vitro* Bone Resorption Assay

BMMs were cultured as previously described. The adherent BMMs were planted onto bovine bone slices (Lushen, Shanghai, China) in a-MEM containing 30 ng/ml M-CSF and 50 ng/ml RANKL. They were also treated with different concentrations of ZG (0 µg/ml, 90 µg/ml, 180 µg/ml) and IGF-1 (100 ng/ml) depending on experimental design. After 10 days, the bovine bone slices were ultrasonically cleaned and cleared with PBS followed by air-drying for 2–3 h. Finally, the samples were subjected to vacuum drying, sprayed with gold, and imaged by SEM (FEI Quanta 200). Quantitative analyses were performed using ImageJ Software.

### *In Vitro* Osteogenesis Assay

Preosteoblast was isolated from calvariae of newborn mice by serial digestion in 1 mg/ml of collagenase type 2. After centrifugation and filtration, preosteoblast was seeded onto six-well plates at a density of 1×10^5^ cells/well in a-MEM supplemented with 10% FBS and 1% penicillin-streptomycin solution. After being cultured to 100% confluence, osteoblast formation was induced through medium replacement with osteogenic differentiation medium (α-MEM supplemented with 50 μg/ml ascorbic acid, 0.1 μM dexamethasone, and 10 mM β-glycerol phosphate). They were also treated with different concentrations of ZG (0 µg/ml, 90 µg/ml, 180 µg/ml) and MHY1485 (10 μM) depending on experimental design. ALP staining (Sigma-Aldrich) was performed on Day 7, and Alizarin red staining was conducted on Day 21, according to the instructions. Both ALP staining and Alizarin red staining were photoed by microscopy (BX53, Olympus) in a blinded manner.

### Western Blot Analysis

The cells were homogenized and lysed in RIPA buffer. Total proteins were extracted by centrifugation at 4°C for 15 min at 13,000 g. Subsequently, total proteins were subjected to 7.5/10/15% SDS-PAGE and transferred onto PVDF membranes (Bio-Rad, CA). The membranes were blocked in 4% nonfat milk, and primary antibodies of TRAP (ab191406, Abcam), CTSK (A1782, ABclonal), TRAF6 (A16991, ABclonal), p-Akt (4060, CST), Akt (4691, CST), p-PI3K (17366, CST), PI3K (4257, CST), mTOR (ab2732, Abcam), p-mTOR (ab109268, Abcam), p-70S6K1 (ab194521, Abcam), Collagen 1 (ab255809, Abcam), RUNX2 (ab236639, Abcam), and β-actin (AC026, ABclonal) were added and incubated at 4°C overnight. HRP-goat anti-rabbit or mouse antibodies (AS003, AS014, ABclonal) were added and incubated at room temperature for 40 min. Finally, the membranes were scanned with chemiluminescence system (Tanon, China).

## Quantitative Real-Time Polymerase Chain Reaction

Total RNA was extracted from the cultured cells using TRIzol (Takara, Japan). cDNA was subsequently reverse transcribed from total RNA using cDNA synthesis kit (Takara, Japan), according to the manufacturer’s instructions. Real-time PCR was carried out by incubation of cDNA, ddH2O, primers, ROX, and SYBR Premix Ex Taq (Takara, Japan). The reagents comprised a total volume of 10 μl per reaction. The samples were subjected to 40 cycles of amplification according to the manufacturer’s instructions. The PCR reactions were carried out in Viia 7 Real-Time PCR System (Applied Biosystems, United States). All data were normalized to β-actin by the 2^(−ΔΔCt) method, and the tests were carried out in triplicate. The sequences of the used primers are indicated in Supplementary Table S1.

### Luciferase Reporter Assays of NF-κB

The BMMs were seeded in 48-well plates at a density of 1.5 × 10^5^ cells/well in a-MEM containing 30 ng/ml M-CSF. Cells were then transfected with pNFκB-TA-luc and pRL-TK responsive luciferase plasmid (Beyotime). After 48 h, the BMMs were treated with or without ZG (90 µg/ml, 180 µg/ml) and RANKL (50 ng/ml) for 2 h. At the end of the time points, cells were lysed, and luciferase activity was measured using the luciferase reporter assay kit (Beyotime) and a luminescence reader (Molecular Devices M3, United States).

### Statistical Analysis

In this study, original data obtained from three or more independent experiments were analyzed by one-way ANOVA with Tukey’s HSD post hoc test and presented as mean ± standard deviation (SD). The results of statistical analysis with *p*-values < 0.05 were considered to be statistically significant (95% confidence interval).

## Results

### Quality Control of Zhuangguguanjie Formulation

ZG is a TCM formulation recorded in the Chinese Pharmacopoeia and approved by the CFDA (Approval No: S20080055). Each batch of ZG is manufactured in strict accordance with national execution standard (WS3-709 (Z-141)-2010Z), and the conformity of main components was identified and compared. We used LC-MS/MS to determine the major components of ZG for every batch experiment. The total positive and negative ion chromatograms of ZG are shown in Supplementary Figures S1, S2. The identification of possible compound names of the main ingredients in ZG is listed in Supplementary Tables S2, S3. The content determination of the main compounds was shown in Supplementary Table S4.

### Oral Administration of Zhuangguguanjie formulation Alleviated Bone Loss *via* Inhibiting Bone Resorption and Promoting Bone Formation in Ovariectomized Mouse Model

**FIGURE 1:**
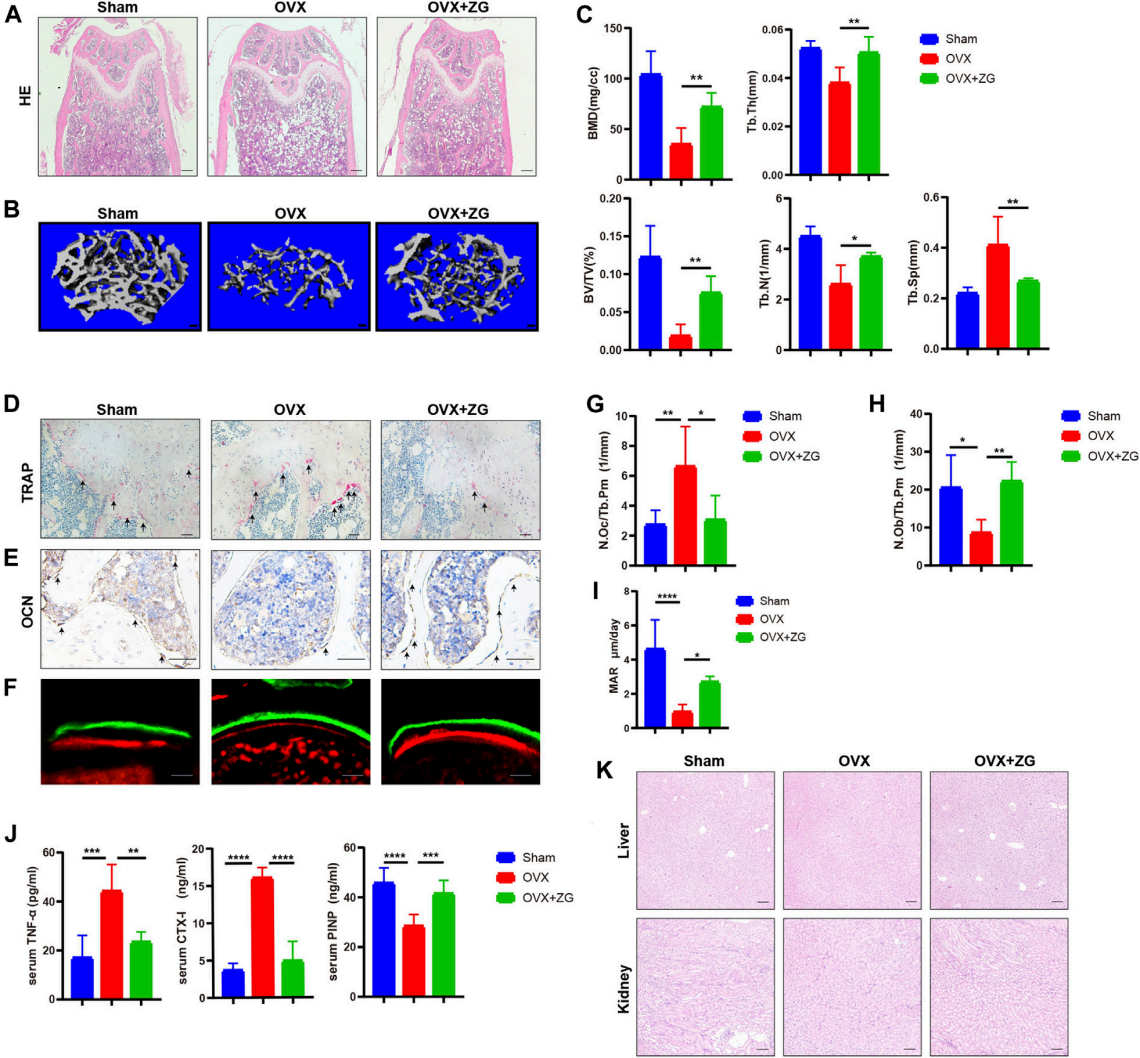
ZG promotes bone formation and inhibits bone resorption to ameliorate ovariectomy-induced bone loss *in vivo*. **(A)** The representative H&E staining images of distal femoral sections from each group. Scale bar = 200 μm. **(B)** The representative reconstructed images of three-dimensional trabecular architecture in distal femur from sham mice without treatment (sham) and ovariectomized mice treated without (OVX) or with (OVX + ZG) ZG obtained by micro-CT examination at 4 weeks after treatment. Scale bar = 100 μm. **(C)** Micro-CT analysis for the architectural parameters of trabecular bone at distal femur including bone mineral density (BMD), bone volume/total volume (BV/TV), trabecular number (Tb.N), trabecular thickness (Tb.Th), and trabecular separation (Tb.Sp) from sham, OVX, and OVX + ZG group. **(D)** The representative TRAP staining images of distal femur sections from each group, scale bar = 100 μm. **(E)** The representative images of immunohistochemical staining for osteocalcin (OCN) at distal femur from each group, scale bar = 100 μm. **(F)** The representative fluorescent micrographs of newly formed bone at distal femur metaphysis during 7-day interval of double labeling with the xylenol (red) and calcein (green) labels in respective groups. Scale bar = 20 μm. **(G–I)** Bone histomorphometric analysis of osteoclast number (N.Oc/Tb.Pm) **(G)**, osteoblast number (N.Ob/Tb.Pm) **(H),** and mineral apposition rate (MAR) **(I)** at distal femur from respective groups. **(J)** The ELISA assay for the serum levels of tumor necrosis factor (TNF-α), cross-linked carboxy-terminal telopeptide of type I collagen (CTX-1), and procollagen I N-terminal propeptide (PINP) in each group. **(K)** The representative H&E staining images of liver and kidney from each group. Scale bar = 200 μm. Notes: Data are presented as mean ± SD, *p < 0.05, **p < 0.01, ***p < 0.001, ****p < 0.0001 by one-way ANOVA with Tukey’s HSD post hoc test, n = 6 per group.

To investigate the therapeutic effects of ZG on osteoporosis, we orally administrated ovariectomized (OVX) mice with ZG at a dosage of 468 mg/kg every day. We observed better organized trabecular micro-architecture with a higher bone mass in OVX + ZG group than that in OVX group after treatment both in HE staining (Figure 1A) and 3D reconstruction (Figure 1B) images. Comparing to the sham-operated group, the micro-CT analysis also showed that administration of ZG significantly increased bone mineral density (BMD), relative bone volume (BV/TV), trabecular thickness (Tb.Th), trabecular number (Tb.N), and reduced trabecular separation (Tb.Sp) in OVX + ZG group (Figure 1C). Consistent with the results in distal femur, ZG also improved the bone mass and micro-trabecular architecture in both femoral neck (Supplementary Figure S3A,B) and proximal tibia (Supplementary Figure S3C,D). We observed lesser osteoclast number by TRAP staining (Figure 1D) and more osteoblast number by OCN immunostaining (Figure 1E) in OVX + ZG than those in OVX group. The quantitative bone histomorphometric analysis further confirmed that osteoclast number (N.Oc/Tb.Pm) decreased (Figure 1G), whereas the osteoblast number (N.Ob/Tb.Pm) increased (Figure 1H) in OVX + ZG compared to these in OVX group, respectively. Finally, we used double labeling with the xylenol (red) and calcein (green) labels to measure newly formed bone at distal femur metaphysis. The fluorescent micrographs suggested that treatment with ZG increased the mineralization of bone matrix (Figure 1F). This result was further confirmed by quantification of MAR (Figure 1I). Subsequently, the serum levels of TNF-α, CTX-1, and PINP were tested by ELISA. Compared with OVX group, ZG reduced the level of serum TNF-α and bone resorption marker CTX-1, while increased the serum level of bone formation marker PINP (Figure 1J). No adverse events in hepatic and renal toxicity were observed during ZG treatment as evidenced that H&E staining of liver and kidney sections displayed no obvious changes among sham, OVX, and OVX + ZG groups (Figure 1K). Our findings indicated that ZG may restrain ovariectomy-induced bone loss by inhibiting osteoclastic bone resorption and promoting osteoblastic bone formation.

### PI3K/Akt/mTOR Pathway Was Identified as the Potential Underlying Mechanism of ZG Action on Osteoporosis by Network Pharmacology-Based Analysis

#### Identification for Potential Targets of Zhuangguguanjie Formulation

The active components of ZG formula comprising multiple herbs, including Radix Angelicae Biseratae, Drynariae Rhizoma, Spatholobus Suberectus Dunn, Myrrha, Aucklandiae Radix, Olibanun, Herba Taxilli, Rehmanniae Radix Praeparata, Dipsaci Radix, and Epimrdii Herba were identified based on TCMSP with the criteria of OB ≥ 30% and DL ≥ 0.18, besides that the effective components of Psoralea corylifolia and Cibotium barometz were screened under setting score ≥20 in the Batman database. The extra verified active elements were added in combination based on the literature data mining, such as naringin, hederagenin, Asperosaponin VI, and so on. At last, the reported active ingredients of ZG were confirmed, and the potential targets were identified based on the Swiss Target Prediction database (Table 1).

**TABLE 1:** Statistical table for basic information of traditional Chinese medicine-composition-target in ZG.

Herb Name	Bioactive Ingredient	Predicted Target
Psoralea corylifolia	29	461
Radix Angelicae Biseratae	9	276
Cibotium barometz	29	480
Drynariae Rhizoma	20	398
Spatholobus Suberectus Dunn	24	509
Myrrha	45	528
Aucklandiae Radix	8	216
Olibanun	8	193
Herba Taxilli	4	153
Rehmanniae Radix Praeparata	5	119
Dipsaci Radix	9	359
Epimrdii Herba	29	447

#### Compound-Target Network Construction

**FIGURE 2:**
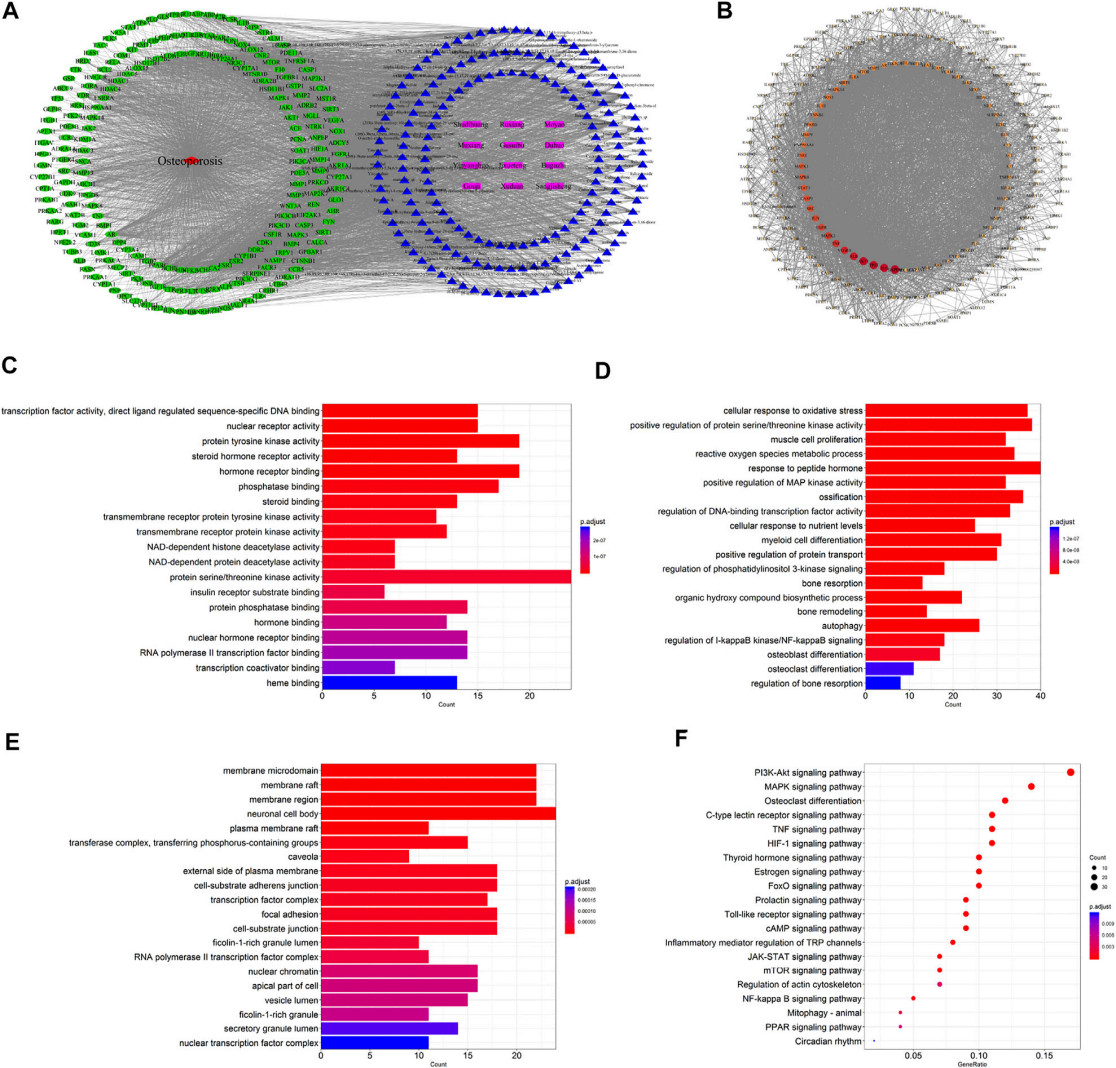
Network pharmacology-based analysis for target genes and underlying pathways of ZG in osteoporosis treatment. **(A)** The drug-bioactive ingredient-target-disease network of ZG acting on osteoporosis based on the interaction of drug (ZG), bioactive ingredients, target genes, and disease. Purple nodes represent the drug; blue nodes represent the bioactive ingredients; green nodes represent the osteoporosis-related targets; red node represents the disease. **(B)** The proteinprotein interaction (PPI) network of overlapping genes between ZG and osteoporosis was obtained from the STRING database and constructed by Cytoscape. The nodes represent the overlapping genes. The edges indicate the interaction among the nodes. The size and colors of the nodes from red to orange to blue were illustrated in descending order of degree values. **(C–E)** The gene ontology (GO) enrichment analysis for overlapping genes between ZG and osteoporosis associated with molecular functions **(C)**, biological processes **(D),** and cellular components **(E)**. The x-axis showing the significant enrichment in the genes counts. The y-axis showing the categories in the GO of the target genes. **(F)** The Kyoto Encyclopedia of Genes and Genomes (KEGG) pathway enrichment analysis of overlapping genes between ZG and osteoporosis.

A total of 1,098 disease targets were obtained from the Disgenet database with “Osteoporosis” as the keyword. Then we generated 212 common targets by taking the intersection of 1,018 drug targets and 1,098 disease targets. The 196 potential active components in ZG and 212 drug–disease common targets were inputted into the Cytoscape software, and the network diagram of “drug-component-target-disease” interaction was drawn (Figure 2A). Among these bioactive components, luteolin exhibited the highest correlation with osteoporosis targets based on the degree value, and the rest were kaempferol, quercetin, stigmasterol, sitosterol, and so on.

#### PPI Network Analysis

The 212 common targets were submitted to the STRING database, which provided the information of predicted interaction, and they were imported in Cytoscape to analyze and construct the PPI network (Figure 2B), in which targets with a higher degree played a momentous role in the correlation. The top 10 targets were GAPDH, IL6, AKT1, TP53, ALB, VEGFA, TNF, MAPK3, EGFR, and JUN, ranked by the degree value.

#### GO Enrichment Analysis

The biological process, cellular composition, and molecular function were selected by GO analysis of 212 common targets. The results of GO showed that the set of overlapping genes was enriched into 197 molecular functions, which were mainly involved in protein serine/threonine kinase activity, protein tyrosine kinase activity, hormone receptor binding, etc (Figure 2C). The set of overlapping genes was enriched into 2748 biological processes including response to peptide hormone, ossification, cellular response to oxidative stress, osteoblast differentiation, osteoclast differentiation, etc (Figure 2D). The set of overlapping genes was enriched into 75 cellular components including neuronal cell body, membrane raft, membrane region, etc (Figure 2E).

#### KEGG Enrichment Analysis

To further explore the potential pathways of ZG on osteoporosis, a total of 162 KEGG pathways were selected by KEGG analysis of 212 common targets. As a result, a bubble diagram with functional enrichment of KEGG pathways was formed (Figure 2F). The result showed that common targets mainly enriched in the PI3K/Akt signaling pathway, MAPK signaling pathway, osteoclast differentiation, mTOR signaling pathway, and PPAR signaling pathway were also included. The PI3K/Akt/mTOR pathway plays an important role in the regulation of both osteoclast and osteoblast differentiation, suggesting that ZG may change the PI3K/Akt/mTOR pathway to affect the balance of bone formation and resorption.

#### Cytotoxicity Study of Zhuangguguanjie Formulation in BMMs and Preosteoblast

We first examined the effect of ZG on viability of BMMs. BMMs were incubating with ZG at concentrations of 0, 30, 60, 90, 180,360, and 720 µg/ml for 24 h. The results showed that cell viability of BMMs was significantly inhibited after ZG treatment at the concentration of 360 µg/ml for 24 h while lower concentrations had no significant effect (Supplementary Figure S4). The same method was used to test cytotoxicity of ZG on preosteoblast. When cultured with ZG for 24 h, the cell viability of preosteoblast was significantly inhibited when the concentration of ZG was greater or equal to 360 µg/ml (Supplementary Figure S5). Therefore, 90 and 180 µg/ml ZG were selected as suitable doses in subsequent experiments to exclude interference from suppression of the proliferation of BMMs and osteoblast.

#### Zhuangguguanjie Formulation Inhibited Osteoclast Differentiation Through the PI3K/Akt Pathway *in vitro*

**FIGURE 3:**
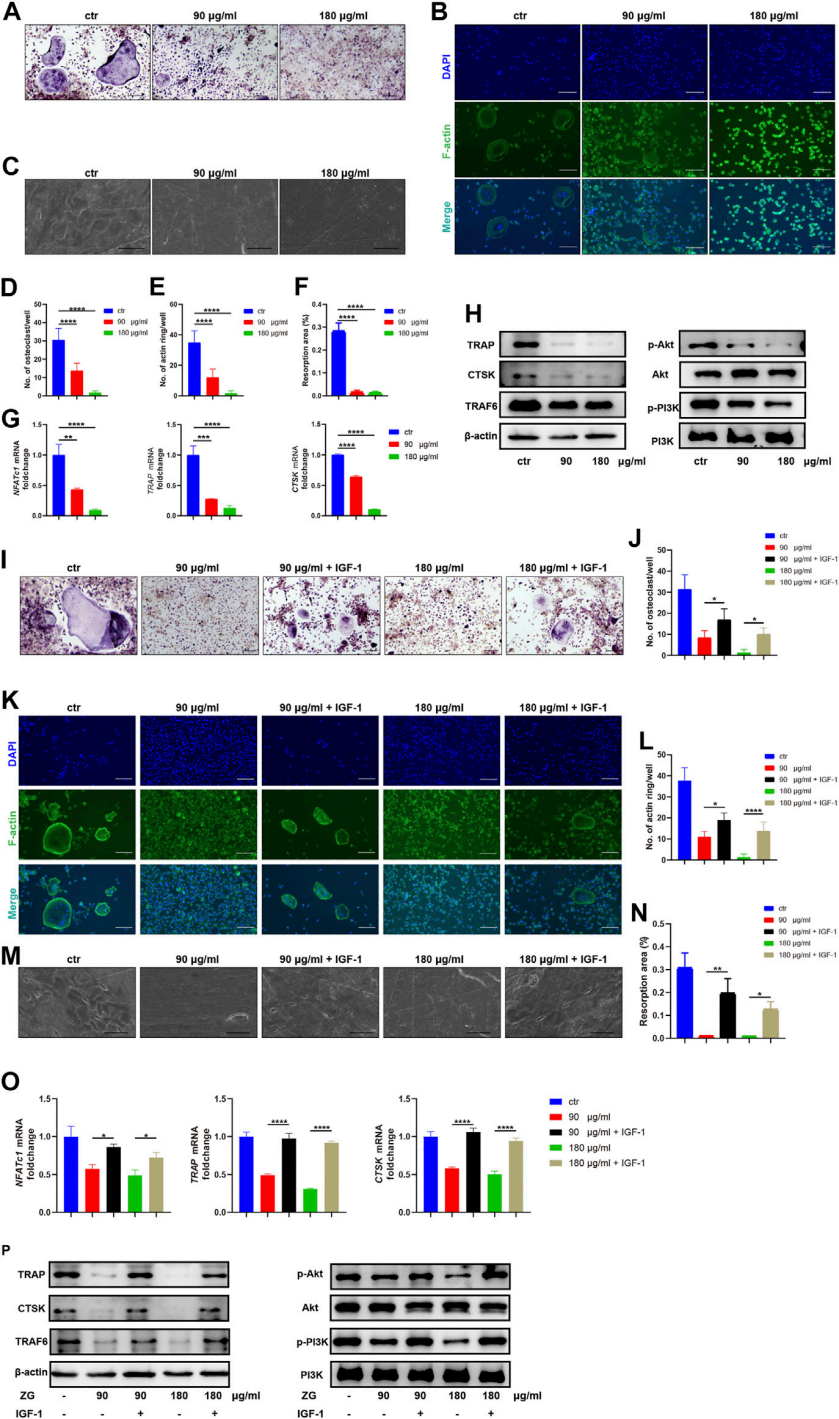
ZG inhibits the PI3K/Akt signaling pathway to reduce osteoclast differentiation *in vitro*. **(A–C)** The representative images of TRAP staining **(A)**, phalloidin staining **(B),** and resorption pits **(C)** for BMMs at day 10 after treatment with vehicle (ctr) and ZG at 90 or 180 μg/ml concentration, respectively. **(A,B)** Scale bar = 100 μm, **(C)** Scale bar = 20 μm. **(D)** The number of multinucleated osteoclasts with TRAP-positive staining per well in indicated groups. **(E)** The number of actin ring per well in indicated groups. **(F)** Quantification for the percentage of resorption pit area relative to total bovine bone slices area in indicated groups. **(G)** Q-PCR analysis for mRNA expression of osteoclast differentiation marker genes including NFATc1, TRAP, and CTSK in BMMs treated with vehicle (ctr) and ZG at 90 or 180 μg/ml concentration, respectively. **(H)** Western blot analysis for protein levels of osteoclast differentiation markers (TRAP, CTSK, and TRAF6) and PI3K/Akt signaling pathway markers (PI3K, p-PI3K, Akt, and p-Akt) in BMMs treated with vehicle (ctr) and ZG at 90 or 180 μg/ml concentration, respectively. **(I,J)** The representative images of TRAP staining **(I)** and quantification of multinucleated osteoclasts **(J)** for BMMs treated with ZG in combination with or without IGF-1. Scale bar = 100 μm. **(K,L)** The representative images of phalloidin staining **(K)** and quantification of actin ring **(L)** for BMMs treated with ZG in combination with or without IGF-1. Scale bar = 100 μm. **(M,N)** The representative images of resorption pits **(M)** and quantification of resorption area **(N)** for BMMs treated with ZG in combination with or without IGF-1. Scale bar = 20 μm. **(O)** Q-PCR analysis for mRNA expression of osteoclast differentiation marker genes including NFATc1, TRAP, and CTSK in BMMs treated with ZG in combination with or without IGF-1. **(P)** Western blot analysis for protein levels of osteoclast differentiation markers (TRAP, CTSK, TRAF6, and β-actin) and PI3K/AKT signaling pathway markers (PI3k, p-PI3K, Akt, and p-Akt) in BMMs treated with ZG in combination with or without IGF-1. Notes: Data are presented as mean ± SD, *p < 0.05, **p < 0.01, ***p < 0.001, ****p < 0.0001 by one-way ANOVA with Tukey’s HSD post hoc test. Each experiment was performed in triplicate and repeated three times independently.

To explore the inhibitory effects of ZG on osteoclast differentiation, we administrated ZG during osteoclast differentiation *in vitro* at the concentration of 90 or 180 μg/ml, which was determined by CCK-8 as the optimal low and high concentration of ZG for *in vitro* administration. After treated with ZG for 24 h, the number of multinuclear TRAP-positive cells was significantly lesser than that in control group (Figures 3A,D). We next used phalloidin staining to assay the formation of actin ring which was an important indicator of maturity during the process of differentiation from macrophages to osteoclasts. Consistently, ZG treatment significantly reduced the number of the newly formed actin rings during osteoclast differentiation in a dose-dependent manner (Figures 3B,E). To further investigate the functional activity of osteoclast on resorption of organic bone matrix and dissolution of inorganic components, we planted osteoclasts onto bovine bone slices to assess osteoblast resorptive ability by measuring the resorption area. Electron microscopy imaging and semi-quantitative analysis showed that the resorptive area on bovine slice was reduced after ZG treatment (Figures 3C,F). In addition, the mRNA levels (Figure 3G) and the protein levels (Figure 3H) of osteoclast differentiation markers, including NFATc1, TRAP, and CTSK, were suppressed by ZG treatment, respectively.

In order to validate the underlying signaling pathway for ZG action on osteoclasts predicted by network pharmacology, we detected the expression changes of key genes in the PI3K/Akt signaling pathway with the highest enrichment index in the network pharmacology. Western blot indicated that ZG reduced the phosphorylation levels of PI3K and Akt (Figure 3H). To further confirm that ZG inhibited osteoclast differentiation *via* the suppression of the PI3K/Akt signaling pathway, we test that whether the PI3K/Akt activator IGF-1 could restrain the inhibitory effects of ZG on the PI3K/Akt signaling pathway. As showed in the images of TRAP staining, the addition of IGF-1 rescued the number of multinuclear TRAP-positive cells which was reduced by ZG (Figures 3I,J). The formation of actin ring was also enhanced after treated with IGF-1 (Figures 3K,L). As one of the intuitive indicators for osteoclast function, IGF-1 significantly recovered the resorptive ability of osteoclast represented by pit formation assay (Figures 3M,N). Meanwhile, the addition of IGF significantly reversed the inhibitory effects of ZG treatment on the expression of osteoclast differentiation genes including *NFATc1*, *TRAP*, and *CTSK* (Figure 3O). Finally, we observed that IGF-1 treatment rescued the ZG-mediated depression of TRAP, CTSK, TRAF6, p-PI3K, and p-Akt in protein levels (Figure 3P). Overall, we confirmed that ZG could inhibit osteoclast differentiation through inhibiting the PI3K/Akt pathway.

#### Zhuangguguanjie formulation Promoted Osteoblast Differentiation Through the mTORC1/S6K1 Pathway *in vitro*

**FIGURE 4:**
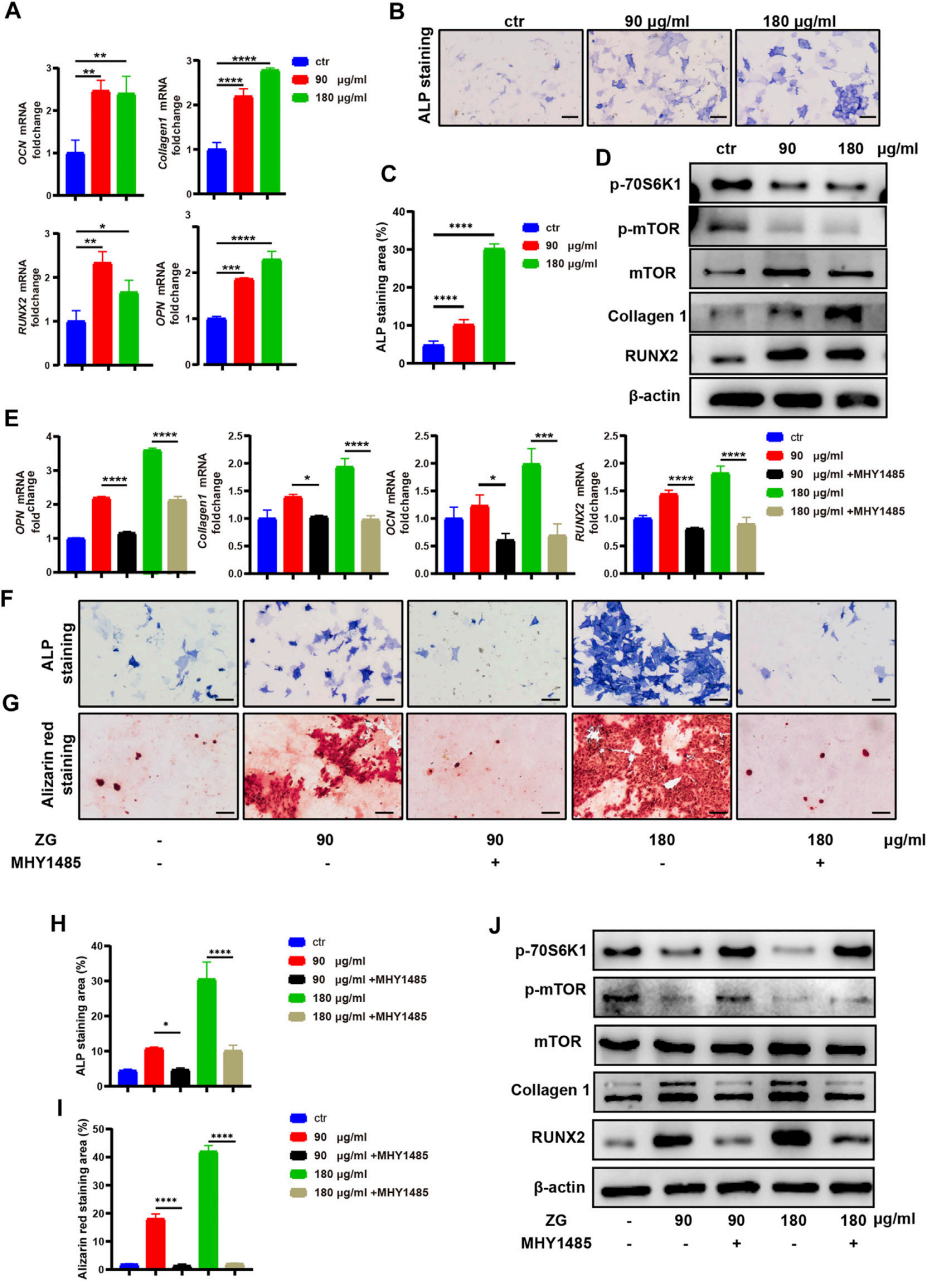
ZG inhibits the mTORC1/S6K1 signaling pathway to promote osteoblast differentiation *in vitro*. **(A)** Q-PCR analysis for mRNA expression of osteogenic differentiation marker genes including osteocalcin (OCN), collagen 1, RUNX2, and osteopontin (OPN) in preosteoblast treated with vehicle (ctr) and ZG at 90 or 180 μg/ml concentration, respectively. **(B)** The representative images of ALP staining for preosteoblast at day 7 after treatment with vehicle (Ctrl) and ZG at 90 or 180 μg/ml concentration, respectively. **(C)** Quantification for the percentage of ALP staining area relative to total area in indicated groups. **(D)** Western blot analysis for protein levels of osteoblastic differentiation markers (collagen1, RUNX2, and β-actin) and mTORC1/S6K1 signaling pathway markers (p-70S6K1, p-mTOR, and mTOR) in preosteoblast treated with vehicle (ctr) and ZG at 90 or 180 μg/ml concentration, respectively. **(E)** Q-PCR analysis for mRNA expression of osteogenic differentiation marker genes including OCN, collagen 1, RUNX2, and OPN in preosteoblast treated with ZG in combination with or without MHY1485. **(F)** The representative images of ALP staining for preosteoblast treated with ZG in combination with or without MHY1485. **(G)** The representative images of alizarin red staining for preosteoblast treated with ZG in combination with or without MHY1485. **(H)** Quantification for the percentage of ALP staining area relative to total area in indicated groups. **(I)** Quantification for the percentage of Alizarin red staining area relative to total area in indicated groups. **(J)** Western blot analysis for protein levels of osteoblastic differentiation markers (collagen1, RUNX2, and β-catenin) and mTORC1/S6K1 signaling pathway markers (p-70S6K1, p-mTOR and mTOR) in preosteoblast treated with ZG in combination with or without MHY1485. Notes: Scale bar = 100 μm. Data are presented as mean ± SD, *p < 0.05, **p < 0.01, ***p < 0.001, ****p < 0.0001 by one-way ANOVA with Tukey’s HSD post hoc test. Each experiment was performed in triplicate and repeated three times independently.

To investigate the effects of ZG on osteoblast differentiation, the primary preosteoblasts isolated from calvaria were treated with vehicle (Ctrl) and ZG at 90 or 180 μg/ml for 24 h, respectively. The optimal low and high concentrations of ZG were determined by CCK-8. We observed that the expression levels of osteogenic differentiation marker genes, including *osteocalcin (OCN)*, *collagen 1*, *RUNX2*, and *osteopontin (OPN)*, in osteoblasts treated with ZG were all obviously higher than those in osteoblast-treated vehicle, respectively (Figure 4A). Following 7 days of osteogenic induction, ZG treatment also enhanced ALP expression, as evidenced by lager positive ALP staining areas in ZG group than that in Ctrl group (Figures 4B,C). The protein levels of osteoblastic differentiation marker including Collagen 1 and RUNX2 were also enhanced with ZG treatment, detected by western blot (Figure 4D). To validate the mTOR signaling pathway as the potential mechanism for ZG action on osteoblasts predicted by network pharmacology, we examined the changes of protein levels of p-mTOR and p-70S6K1 in preosteoblast during ZG treatment by western blot. We found that the protein level of p-mTOR and p-70S6K1 in preosteoblast significantly decreased after ZG treatment. To further confirm whether ZG promotes osteoblast differentiation *via* inhibiting the mTOR/S6K1 pathway, we respectively added the mTORC1 activator MHY1485 in combination with or without ZG into cell culture as described in experimental design. Indeed, we demonstrated that MHY1485 supplement obviously attenuated the expression levels of osteogenic genes (Figure 4E), the ALP activity (Figures 4F,H), and the capability of extracellular matrix mineralization (Figures 4G,I) in osteoblasts treated with ZG, whereas depressed the protein levels of p-mTOR and p-70S6K1 in osteoblasts treated with ZG (Figure 4J). Thus, our findings confirmed that ZG promotes osteoblastic differentiation through inhibiting the mTOR/S6K1 pathway, and the process could be restrained by mTORC1 activator MHY1485.

## Discussion

During the development of osteoporosis, the balance between osteoclast-mediated bone resorption and osteoblast-mediated bone formation was disrupted (Rodan and Martin, 2000). Multiple targets and pathways were involved in the pathological process of osteoporosis, leading to lower efficacy of single-target drugs than expected (Yang et al., 2020). TCM was considered as an appropriate treatment strategy due to its advantage of multi-components targeting multiple genes involved in the pathway of osteoporosis (Zhang ND. et al., 2016). There was a long history of TCM for treating osteoporosis with remarkable curative effect, while the underlying mechanism still remained unknown (Zhao et al., 2021). Network pharmacology provided visual analysis of drug components, disease targets, and pathways of action, which was helpful to explore the potential mechanism of TCM compounds (Zhou et al., 2020). This study aims to verify the efficacy and mechanism of ZG in the treatment of osteoporosis through network pharmacology and experiments *in vivo* and *in vitro*.

As the ovariectomized mouse model has widely been used to mimic clinical features of postmenopausal osteoporosis, we orally administrated OVX mice with ZG at dosage of 468 mg/kg every day according to dosage conversion formula based on body surface area (Reagan-Shaw et al., 2008). Both H&E staining and micro-CT analysis showed higher bone mass (BMD and BV/TV) and better organized trabecular micro-architecture (Tb.N, Tb.Th, and Tb. Sp) in OVX after ZG treatment than those parameters in OVX mice without treatment. Thus, it revealed that ZG could exert beneficial effect on OVX mouse model. During the process of postmenopausal osteoporosis, the balance between bone formation and bone resorption was disrupted, in which the bone resorption was over-activated and greater than bone formation, resulting in progressive bone loss (Kim and Koh, 2019). In our study, bone histomorphometric analysis suggested that ZG could not only increase the number of osteoblast and promote the mineralization of bone matrix but also reduce osteoclast differentiation and finally prevent bone loss in OVX mouse model. Consistent with previous findings, ZG also reduced the level of serum bone resorption marker CTX-1, while increased the serum level of bone formation marker PINP in OVX mice compared with those in untreated OVX mice.

Since ZG could affect both osteoclastic bone resorption and osteoblastic bone formation *in vivo*, we further explored the potential action mechanism using network pharmacology, which could facilitate exploring the potential interaction mechanism between drug components, diseases, and molecular targets based on network visualization. In the present study, we identified the active components in ZG and drawn the network diagram of “drug–component–target–disease” interaction. A total of 212 drug–disease common targets were predicted. The mutual regulation of various targets creates the occurrence and development of biological processes. PPI network analysis demonstrated the priority of interactions between targets. Targets such as GAPDH, Akt, JUN, and mTOR showed a more important position in the process of affecting other targets. However, a single target with higher influence in PPI network does not necessarily mean that it will participate in the best pathway which common targets enriched. After GO enrichment analysis, we found that these overlapping genes were enriched into 2,748 biological processes including response to peptide hormone, ossification, cellular response to oxidative stress, osteoblast differentiation, osteoclast differentiation, and so on. Further KEGG analysis showed that common targets mainly enriched in the PI3K/Akt signaling pathway, MAPK signaling pathway, mTOR signaling pathway, and PPAR signaling pathway. Combined with the results of KEGG and PPI network, PI3K/Akt and mTOR signaling pathways were predicted to be the possible targets for ZG to exert its anti-osteoporosis effect. It was reported that the PI3K/Akt signaling pathway was responsible for activation of osteoclast differentiation (Moon et al., 2012) and enhancement of osteoclastic bone resorption (Zhao et al., 2020), whereas the mTOR signaling pathway contributed to proliferation of osteoblast and participated in regulation of osteoblast differentiation (Dai et al., 2017). Thus, it was indicated that ZG may promote osteoblastic bone formation and inhibit osteoclastic bone resorption *via* above-predicted molecular pathways. These results provided a basis for us to further explore and validated the predicted mechanism for ZG acting on osteoporosis.

As the progressive bone loss in postmenopausal osteoporosis is mainly due to overactivation of osteoclast (Eastell et al., 2016), ZG may inhibit osteoblastic bone resorption *via* predicted the PI3K/Akt pathway. To validate above findings in animal study and network pharmacology, we used bone marrow mononuclear macrophages to induce osteoclast differentiation *in vitro* and subsequently examine the effects of ZG on osteoclast differentiation. The results proved that ZG could significantly reduce the number of TRAP-positive staining osteoclasts and the formation ability of actin rings. Meanwhile, the mRNA expressions of osteoclast differentiation-related genes such as *TRAP*, *CTSK*, and *NFATc1* were inhibited, and the protein levels of TRAP, CTSK, and TRAF6 in osteoblasts were also decreased by ZG treatment *in vitro*. These results suggested that ZG could inhibit the differentiation and function of osteoclast.

The PI3K/Akt pathway plays an important role in cell proliferation and differentiation (Mosca et al., 2012). Phosphatidylinositol 3-kinase (PI3K) is an intracellular phosphatidylinositol kinase. Various growth factors and signal transduction complexes could activate receptor tyrosine kinases, resulting in autophosphorylation of PI3K. The phosphorylated PI3K further binds to the protein kinase B (Akt) binding site and activates Akt through phosphorylation (Chan and Tsichlis, 2001). Phosphorylated Akt has a variety of cellular functions, including regulation of metabolism, proliferation, survival, transcription, and protein synthesis (Hoxhaj and Manning, 2020). Previous studies had shown that RANKL and M-CSF, which were necessary for osteoclast differentiation, could activate the PI3K/Akt signaling pathway, and activated Akt could then induced osteoclast differentiation through GSK3β/NFATc1 signaling cascade (Moon et al., 2012). The results showed that ZG significantly reduced the levels of phosphorylated PI3K and phosphorylated Akt. The addition of PI3K/Akt activator IGF-1 reversed the inhibition of ZG on osteoclast differentiation and bone resorption ability and restored the expression levels of related genes and proteins. Combined with the experimental results *in vivo*, it was verified that ZG could inhibit osteoclast differentiation by inhibiting the PI3K/Akt pathway.

Although the relative/absolute decreased osteoblastic bone formation was equally contributed to the bone loss in senile/postmenopausal osteoporosis, most of the current therapies for osteoporosis focus on bone resorption, and there is lack of therapeutic strategies to promote bone formation for osteoporosis treatment (Brommage, 2020). Our experimental results *in vivo* showed that ZG up-regulated the expression of osteogenic markers during osteoporosis, increased the number of osteoblast, and promoted the mineralization of new bone. Network pharmacological analysis also predicted that ZG might affect the differentiation process of osteoblast. To validate the findings in animal study and network pharmacological analysis, we evaluated the effects of ZG on the differentiation of primary osteoblast from calvariae of newborn mice. The data demonstrated that different concentrations of ZG could promote the expression of ALP during induced differentiation of preosteoblast, which was a specific marker of osteoblast maturation. ZG also promoted the expression of mRNA and protein levels of osteogenic markers such as OCN and RUNX2. In short, ZG enhanced osteoblastic differentiation of preosteoblast.

Network pharmacology predicted that the mTOR pathway may be the target pathway of ZG functioning on osteoblasts. Mammalian target of rapamycin (mTOR) was an evolutionarily conserved protein kinase including mTORC1 and mTORC2 protein complexes with different structures and functions. S6 kinase 1 (S6K1) was a downstream target and phosphorylated substrate of mTORC1 (Holz et al., 2021). The mTORC1/S6K1 pathway played an important role in the regulation of cell metabolism, autophagy, and proliferation (Liu and Sabatini, 2020). In different studies, the mTORC1/S6K1 pathway showed opposite results in osteogenic function. Recent studies had shown that mTORC1/S6K1 promoted the proliferation of osteoblast but down-regulated the expression of RUNX2 through activation of the Notch pathway during osteoblast differentiation, thereby inhibiting osteoblast differentiation (Huang et al., 2015). Our results showed that ZG inhibited the phosphorylation levels of mTORC1 and S6K1. Meanwhile, MHY1485, an activator of the mTORC1/S6K1 pathway, could restore the phosphorylation of mTORC1 and S6K1 and subsequently reverse the enhancement effects of ZG on osteogenic differentiation. Our findings could prove that ZG promotes osteogenic differentiation by inhibiting the mTORC1/S6K1 pathway.

There were also many other pathways that have been screened by network pharmacology as potential targets of ZG for osteoporosis, such as the NF-κB signaling pathway. NF-κB signaling was considered to be the first event in early osteoclast development from precursors. As the key factor of osteoclast differentiation, RANKL could activate the NF-κB pathway and thus release the inhibition of NFATc1 to positively regulate osteoclast formation and functions (Chen et al., 2019). By constructing the luciferase reporter of NF-κB, we found that ZG could also reduce the nuclear translocation of NF-κB in BMMs stimulated by RANKL (Supplementary Figure S6), suggesting that ZG may also inhibit osteoclast differentiation and treat osteoporosis *via* inhibiting the NF-κB pathway. There were still many potential pathways that may be involved in the treatment of osteoporosis by ZG, which need more attention in future studies.

We studied ZG as a whole herb formulation consisting of many components by network pharmacology and experimental verification. It would be needed to further integrate active components of metabolomics determination with the bioavailability and medicinal properties of these components for better analysis of network pharmacology. This will help us to improve the beneficial anti-osteoporosis effects and avoid side effects of ZG herb formulation for further clinical application. In addition, our study mainly used OVX mice to simulate postmenopausal osteoporosis. In future, other types of osteoporotic animal models would be carried out to determine whether ZG still has significant efficacy for other clinical types of osteoporosis, such as senile osteoporosis and secondary osteoporosis.

Overall, ZG excreted beneficial effects on preventing bone loss in osteoporosis. Based on current mechanism, exploration data from experiments *in vivo* and *in vitro* as well as network pharmacological analysis, ZG could inhibit the PI3K/Akt signaling pathway to reduce osteoclastic bone resorption as well as hamper the mTORC1/S6K1 signaling pathway to promote osteoblastic bone formation. Thus, ZG can be used as an appropriate clinical strategy for the treatment of osteoporosis.

## Data Availability

The original contributions presented in the study are included in the article/Supplementary Material; further inquiries can be directed to the corresponding authors.
