# Supplementary material for: Network Pharmacology-Based Strategy for the Investigation of the Anti-Osteoporosis Effects and Underlying Mechanism of Zhuangguguanjie Formulation
Source: Front Pharmacol. 2021 Oct 1;12:727808. doi: 10.3389/fphar.2021.727808 (PMC8517248; doi:10.3389/fphar.2021.727808)
Supplement: Supplementary file 3 [file Table1.DOCX]

**Table S1**. Primer Sequences used for qPCR.

| **Primer** | **Forward(5’-3’)** | **Reverse(3’-5’)** |
| --- | --- | --- |
| *Collagen 1* | GGGGCAAGACAGTCATCGAATACA | GTGGAGGGAGTTTACACGAAGCAG |
| *OCN* | GGACCATCTTTCTGCTCACTCTG | GTTCACTACCTTATTGCCCTCCTG |
| *OPN* | ACAGCCTGCACCCAGATCCTATA | CGTCAGATTCATCCGAGTCCACA |
| *RUNX2* | TGCACCTACCAGCCTCACCATAC | GACAGCGACTTCATTCGACTTCC |
| *NFATc1* | TAGCATTACTCATTGCCTGATTCCTG | TCCATTCATTAGCATCGGTGTTCT |
| *CTSK* | GAAGAAGACTCACCAGAAGCAG | TCCAGGTTATGGGCAGAGATT |
| *TRAP* | GATGCCAGCGACAAGAGGTT | CATACCAGGGGATGTTGCGAA |
| *β-actin* | TTTCCAGCCTTCCTTCTTGGGTAT | TGTTGGCATAGAGGTCTTTACGG |
