# Supplementary material for: Network Pharmacology-Based Strategy for the Investigation of the Anti-Osteoporosis Effects and Underlying Mechanism of Zhuangguguanjie Formulation
Source: Front Pharmacol. 2021 Oct 1;12:727808. doi: 10.3389/fphar.2021.727808 (PMC8517248; doi:10.3389/fphar.2021.727808)
Supplement: Supplementary file 4 [file DataSheet1.PDF]

Supplementary materials

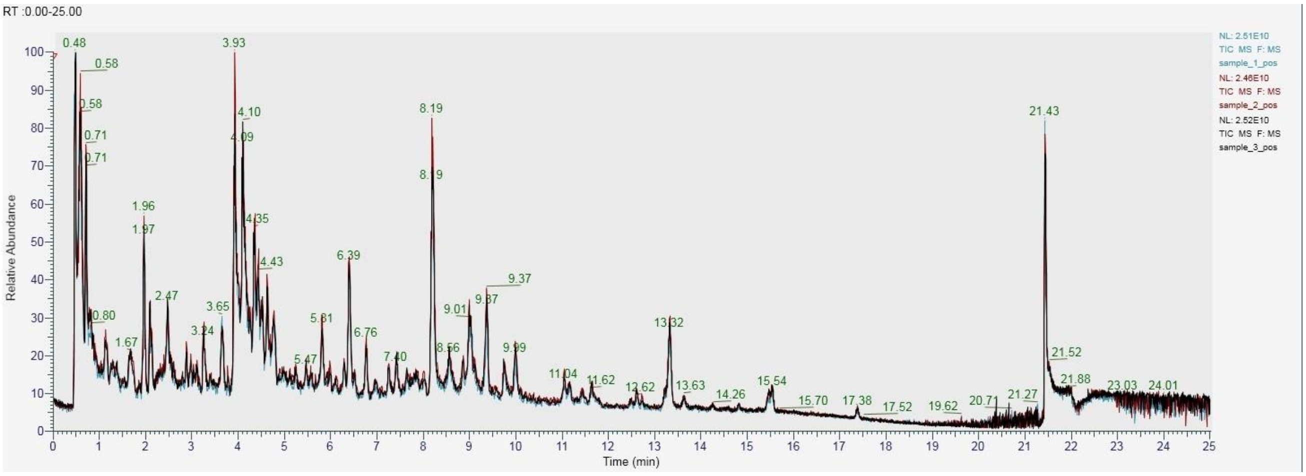

Figure S1 Total ion chromatogram (TIC) of ZG in positive ion mode.

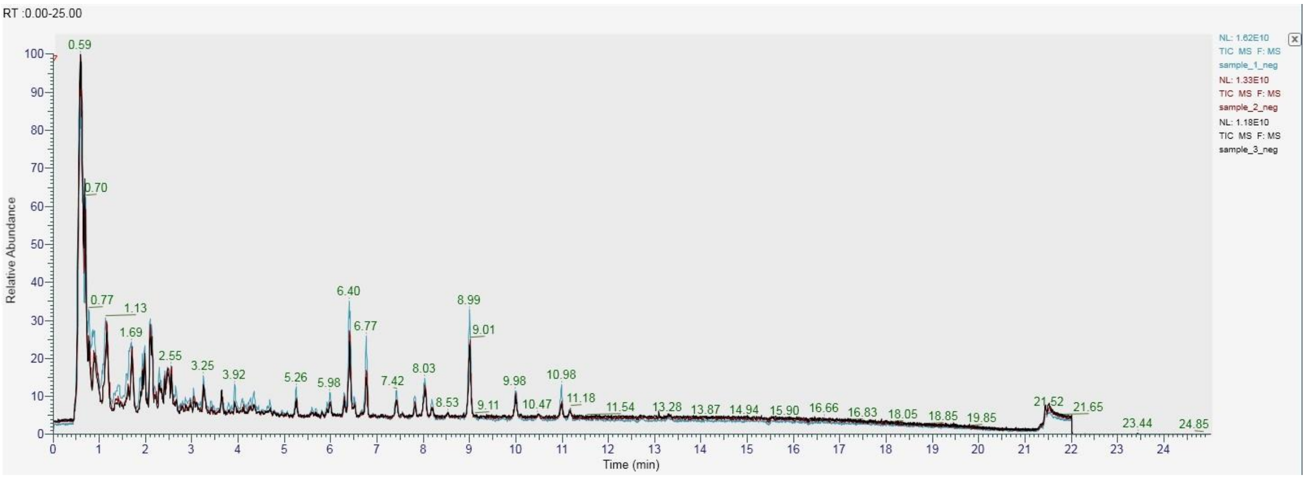

Figure S2 Total ion chromatogram (TIC) of ZG in negative ion mode.

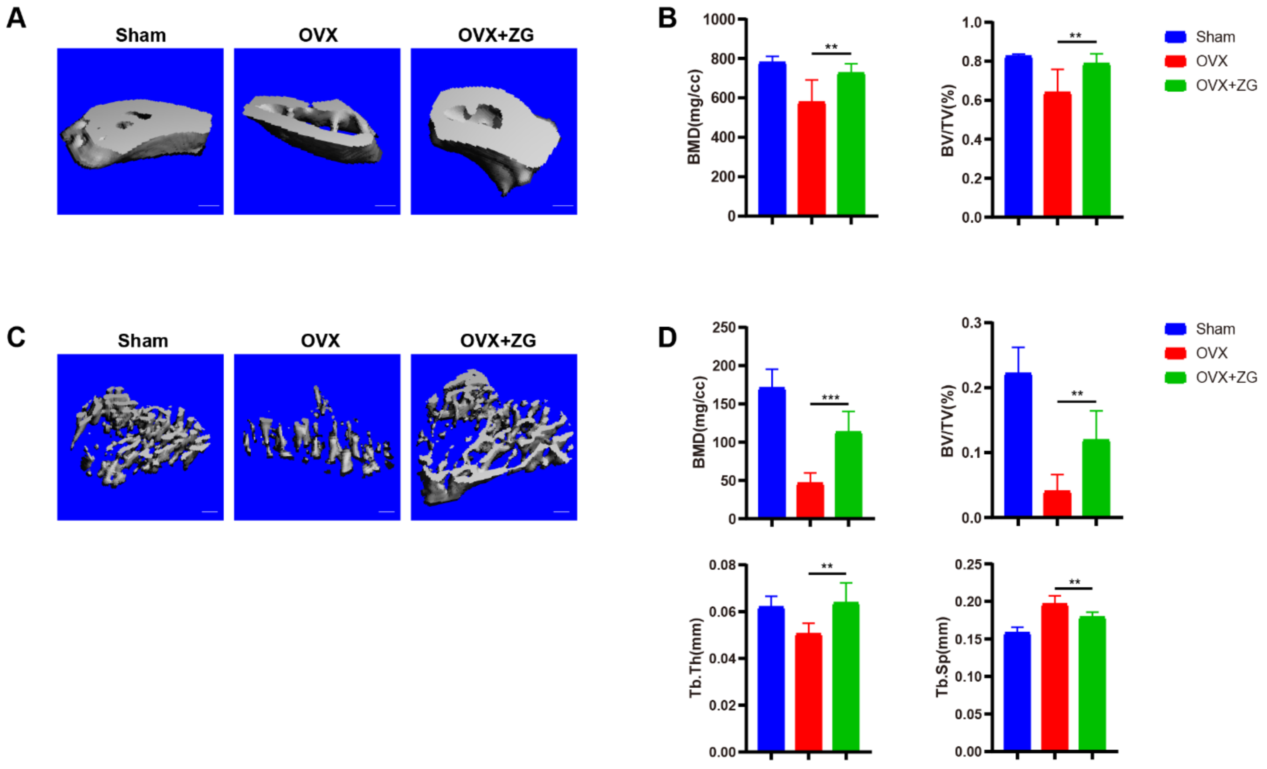

Figure S3 ZG ameliorates ovariectomy-induced bone loss *in vivo.* **(A)** The representative reconstructed images of three-dimensional architecture in femoral neck from sham mice without treatment (Sham) and ovariectomized mice treated without (OVX) or with (OVX+ZG) ZG obtained by micro-CT examination at 4 weeks after treatment. Scale bar=200μm. **(B)** Micro-CT analysis for the architectural parameters of femoral neck including bone mineral density (BMD) and bone volume/total volume (BV/TV) from Sham, OVX and OVX+ZG group. **(C)** The representative reconstructed images of three-dimensional architecture in proximal tibia from Sham, OVX and OVX+ZG group. Scale bar=200μm. **(D)** Micro-CT analysis for the architectural parameters of trabecular bone at proximal tibia including bone mineral density (BMD), bone volume/total volume (BV/TV), trabecular thickness (Tb.Th), and trabecular separation (Tb.Sp) from Sham, OVX and OVX+ZG group. Notes: Data are presented as mean ±SD, $^*P<0.05$, $^{**}P<0.01$, $^{***}P<0.001$ by one-way ANOVA with Tukey's HSD post hoc test, n = 6 per group.

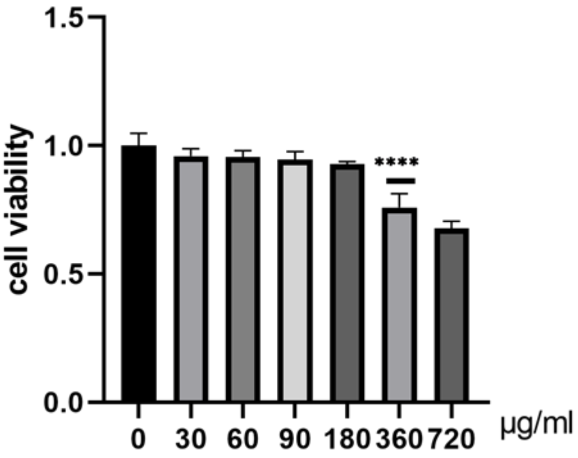

Figure S4 The viability of BMMs with different concentrations of ZG assessed by CCK-8. Data are presented as mean ±SD, ****P<0.0001 by one-way ANOVA with Tukey's HSD post hoc test. The experiment was performed in triplicate and repeated three times independently.

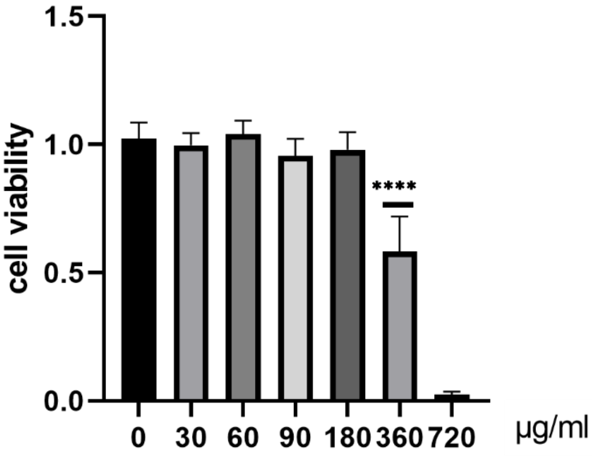

Figure S5 The viability of preosteoblast with different concentrations of ZG assessed by CCK-8. Data are presented as mean ±SD, ****P<0.0001 by one-way ANOVA with Tukey's HSD post hoc test. The experiment was performed in triplicate and repeated three times independently.

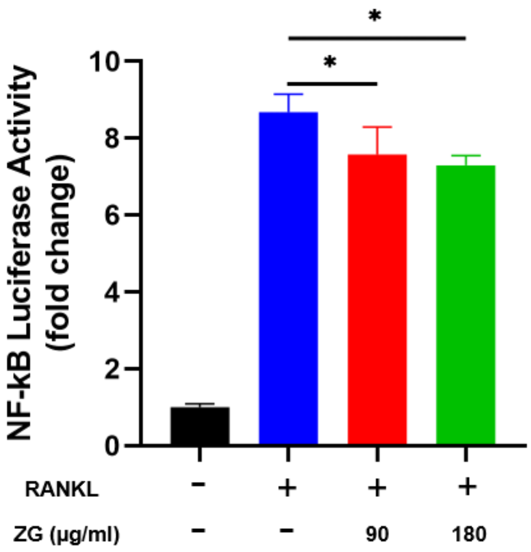

Figure S6 The transcriptional activity of NF-κB detected by luciferase assay. Data are presented as mean ±SD, *P<0.05 by one-way ANOVA with Tukey's HSD post hoc test. The experiment was performed in triplicate and repeated three times independently.
